# Stochastic epigenetic mutation profiles as biomarkers of clinical activity in juvenile idiopathic arthritis: a multi-omic machine learning approach for gene prioritization

**DOI:** 10.1186/s10020-025-01348-6

**Published:** 2025-09-25

**Authors:** Giacomo Cavalca, Matteo Vergani, Davide Cangelosi, Alessandro Consolaro, Marco Gattorno, Angelo Ravelli, Jane Munro, Boris Novakovic, Anna Duncan, Paolo Uva, Giovanni Fiorito

**Affiliations:** 1 University of Bologna, Bologna, Italy; 2 Clinical Bioinformatics Unit, IRCCS Istituto Giannina Gaslini, Genoa, Italy; 3 Rheumatology and Autoinflammatory diseases, IRCCS Istituto Giannina Gaslini, Genoa, Italy; 4 Pediatric and Rheumatology Clinic, IRCCS Istituto Giannina Gaslini, Genoa, Italy; 5 Infection, Immunity and Global Health Theme, Murdoch Children’s Research Institute, Parkville, VIC 3052 Australia; 6 Department of Paediatrics, University of Melbourne, Parkville, VIC 3052 Australia; 7 Royal Children’s Hospital Melbourne, Parkville, VIC 3052 Australia

**Keywords:** Juvenile idiopathic arthritis, DNA methylation, Stochastic epigenetic mutations, Gene expression, Multi-omic, Machine learning

## Abstract

**Background:**

Juvenile idiopathic arthritis (JIA) is a rare autoimmune disease arising from a complex interplay between genetic and environmental factors. Epigenetic modifications such as DNA methylation (DNAm) have been described as potential mediators in gene-environment interactions, contributing to immune system dysregulation. Emerging evidence suggests that DNAm profiles also predict therapeutic responses in autoimmune diseases. This study aims to identify epigenetic biomarkers and epigenetic-driven gene expression changes associated with JIA clinical activity.

**Methods:**

We reanalyzed a publicly available dataset of 44 JIA patients, with whole-genome DNAm and gene expression from CD4 + T cells measured at two points: at anti-TNF therapy withdrawal (T_0_) and eight months later (T_end_). At T_end_, 30 patients maintained inactive disease (ID) while 14 did not (NO ID). We investigated differences between ID and NO ID patients in the epigenetic mutation load and various epigenetic clocks through linear regression models, and prioritized genomic regions with significantly higher number of epimutations in NO ID patients through machine learning.

**Results:**

We found a higher mutation load in NO ID than ID patients, both at T_0_ and at T_end_, with the differences at T_end_ reaching statistical significance (*p* = 0.02). In contrast, we found no evidence of association between epigenetic clocks and JIA clinical activity. Using a multi-omic approach, we identified a List of candidate epigenetically-driven differentially expressed genes, 80 up-regulated and 77 down-regulated, in NO ID patients. Finally, comparing our candidate gene list with the Connectivity Map database, we identified new candidate potential therapeutic targets. Key findings were validated in independent datasets: DNAm profiles from CD4 + T cells (56 JIA patients, 57 controls) and transcriptomic data from PBMCs of JIA patients with active or inactive disease, confirming dysregulation of pathways such as *TNF-α signaling via NF-kB* and *TGF-β signaling* among others.

**Conclusions:**

We described a significant association of epigenetic mutations with JIA clinical activity, indicating that epigenetic changes might precede clinical symptoms and may serve as biomarkers for early disease monitoring. Further, our results shed light on biomolecular mechanisms of JIA, supporting the development of more effective treatments.

**Supplementary Information:**

The online version contains supplementary material available at 10.1186/s10020-025-01348-6.

## Background

Juvenile idiopathic arthritis (JIA) is a broad term that refers to a group of arthritis conditions of unknown origin, characterized by onset before the age of 16 and lasting for more than six weeks (Ravelli and Martini 2007; Martini et al. 2022). JIA is believed to arise from a complex interplay between genetic and environmental factors (Koker et al. 2022). Epigenetic mechanisms, particularly DNA methylation (DNAm), have been described as mediators in gene-environment interactions, potentially contributing to autoimmune disease etiology (Meyer et al. 2015).

Emerging evidence suggests that specific DNAm profiles could serve as biomarkers for predicting therapeutic responses in autoimmune diseases, including JIA (Li et al. 2021).

Several studies show that inhibitors of tumor necrosis factor (anti-TNF) treatment combined with methotrexate significantly increase JIA remission rates. However, a high percentage of patients relapse after treatment withdrawal (Lovell et al. 2000), and patients maintaining inactive disease (ID) are often clinically indistinguishable from those who relapse. Therefore, understanding the mechanisms of disease remission is essential for better clinical decision-making.

Traditional approaches in epigenome-wide association studies (EWAS), which commonly use linear models to identify differentially methylated regions between two groups, may overlook critical biomolecular mechanisms driven by rare epigenetic mutations. Gentilini et al. proposed a methodological approach to identify rare stochastic epigenetic mutations (SEM) (Gentilini et al. 2015) not shared among subjects. Accordingly, the total number of SEMs for each individual was defined as epigenetic mutation load (EML) (Yan et al. 2020). SEMs and EML have been proposed as biomarkers for the cumulative DNA damage resulting from endogenous and exogenous exposures throughout life (Fiorito et al. 2021). Previous literature shows that they increase exponentially during aging (even in physiological conditions) and are significantly associated with higher risk of complex disease in prospective studies, as well as with immune system related disorders (Fiorito et al. 2019).

Additionally, other DNAm-based biomarkers of biological aging such as epigenetic clocks (Horvath 2013; Duan et al. 2022), are recently gaining popularity as robust biomarkers of the general health status and have been extensively used for predicting longevity and aging-related diseases proving to be promising biomarkers for risk stratification (Margiotti et al. 2023). Since the development of the original epigenetic clock by Horvath (Horvath 2013), several other clocks have been introduced. The literature distinguishes between first-generation clocks, trained to predict chronological age, and next-generation clocks, which are trained on outcomes such as mortality risk or biomarkers of healthy aging (Chervova et al. 2024). Despite differences in their training targets, all epigenetic clocks operate on a similar principle: they estimate epigenetic age using a small set of CpG sites; then, the residuals from regressing epigenetic age on chronological age provide insights into an individual’s biological aging status, where positive residuals indicate accelerated aging and vice versa. In adult rheumatoid arthritis (RA), higher biological aging values, measured with epigenetic clocks, have been observed in RA patients compared to healthy controls (Mukherjee and Harrison 2024). In the pediatric population, epigenetic clocks are less frequently studied, largely because most of these biomarkers are trained to predict mortality in adult populations (Mukherjee and Harrison 2024). However, existing literature provides some evidence of an association between accelerated epigenetic aging and neurodevelopmental disorders in prospective studies (Dammering et al. 2021; Gomaa et al. 2022). In this work, we investigated differences in EML and various epigenetic clocks in JIA patients comparing those who did not maintain inactive disease (NO ID) status eight months after the withdrawal of anti-TNF therapy with those who sustained inactive disease (ID) status during the observation period. We aim to identify potential biomarkers that are predictive of prognosis as well as those that change in response to disease reactivation. Further, we applied various machine learning approaches to prioritize genomic regions and genes with a higher number of SEMs in NO ID compared to ID JIA patients, with the aim of identifying potentially dysregulated pathways and molecular mechanisms, and consequently novel therapeutic target candidates.

## Methods

### Dataset and workflow description

We downloaded a publicly available dataset of 68 (56 polyarticular and 12 extended oligoarticular) JIA patients from the GEO repository (GSE89253) (Spreafico et al. 2016). Whole-genome DNAm and gene expression from CD4^+^ T cells were measured with the Illumina HumanMethylation450 Bead Chip array and the Illumina HumanHT-12 v4.0 expression Bead Chip array, respectively, at two time points: at the time of anti-TNF therapy withdrawal (T_0_), when all patients had inactive disease (ID), and eight months after the baseline (T_end_), with 14 out of 68 patients not maintaining inactive disease (NO ID) status (Table 1).

**Table 1:** Characteristics of the study population stratified by clinical activity at T_end_. No significant differences in the distribution of sex by clinical activity (chi-squared test *p* = 0.99). Statistically significant differences in the distribution of age by clinical activity (T test *p* = 0.04). No significant differences in the distribution of disease form by clinical activity (chi-squared test *p* = 0.69)

	ID	NO ID
# of JIA patients	30 (68%)	14 (32%)
# of Females (%)	21 (70%)	10 (71%)
Age at T0: mean (std. dev.)	9.91 (3.55)	13.42 (3.69)
Disease form: - # of oligoarticular extended (%) - # of polyarticular (%)	5 (17%) 25 (83%)	1 (7%) 13 (93%)

Twenty-four out of 68 JIA patients experienced acute disease flares at T_end_. For these analyses, we excluded “flare” patients based on prior observations from the original study by Spreafico et al. (Spreafico et al. 2016), which reported epigenetic similarities between ID and flare patients. Instead, we focused our comparison on ID versus NO ID patients to increase the likelihood of identifying biomarkers of disease reactivation at an early stage. Additional details about wet lab methods are available in the original publication (Spreafico et al. 2016). DNAm data were loaded as fluorescence intensities of methylated sites out of the total fluorescence intensity (beta values), setting all the CpGs with detection p-value higher than 0.01 as missing. We filtered CpGs and samples with a call rate lower than 95% and imputed the few remaining missing values with the median across all patients. After quality control and filtering procedure, our dataset consisted of 338,688 CpGs, in 88 samples (no samples were excluded based on quality control procedures).

Similarly, we followed the same pre-processing procedure for transcriptomic data, removing transcripts and samples with more than 5% missing values, and median imputing the remaining missing ones. After these steps, we analyzed 10,243 transcripts while no samples were removed.

We performed a preliminary descriptive analysis using uniform manifold approximation and projection (UMAP) (McInnes 2018), considering DNAm and transcriptomic data separately, to investigate the presence of clusters of patients with aberrant epigenetic or transcriptomic profiles. Any cluster or outlier was identified in JIA patients (Figure S1 and Figure S2).

### Study design

**Fig. 1:**
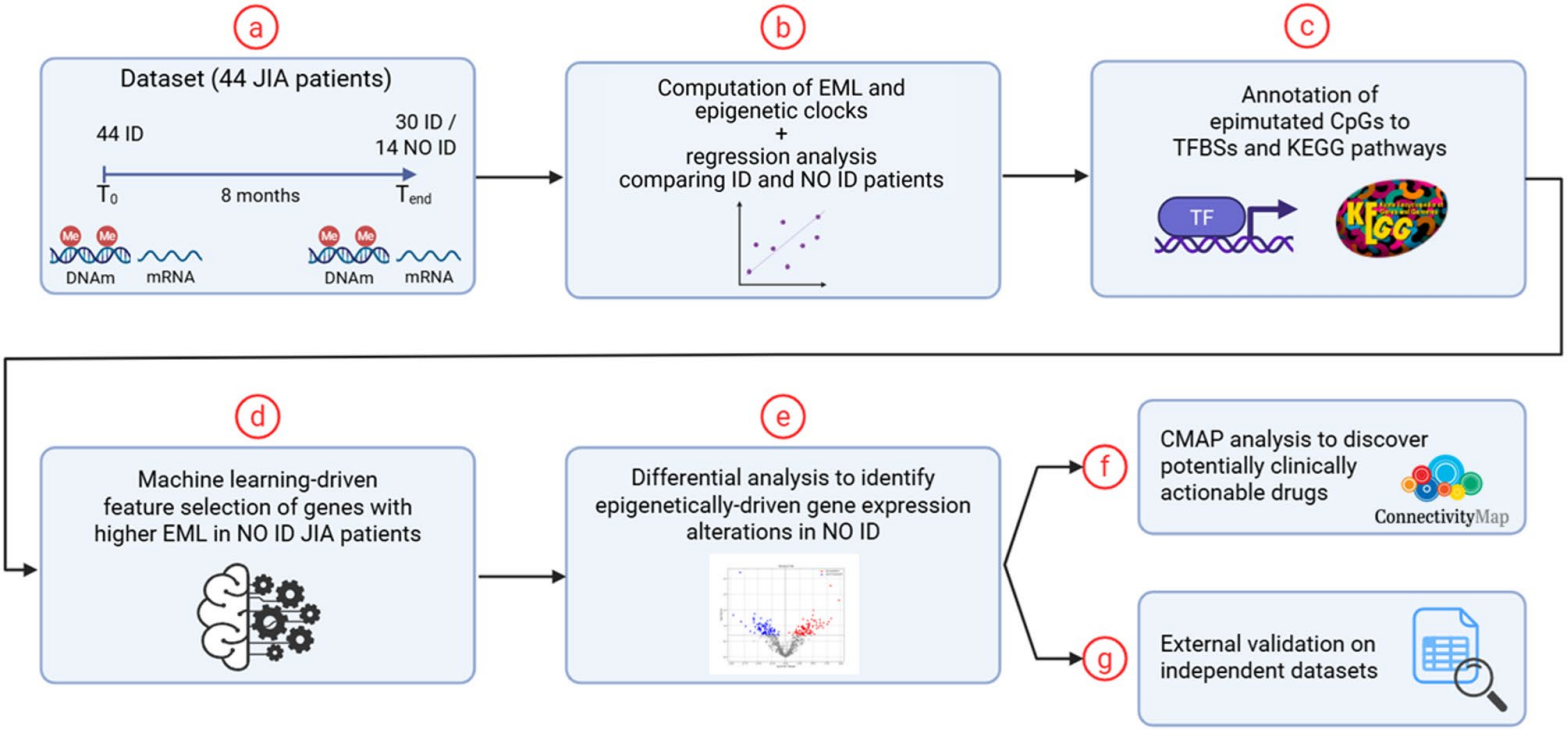
**–** Schematic representation of the workflow: (**a**) Description of the dataset including DNAm and mRNA whole-genome data from 68 JIA patients at two time points; (**b**) Identification of SEMs, computation of EML, and 19 epigenetic clocks for each patient. Regression models to investigate differences between ID and NO ID patients; (**c**) Annotation of epimutated CpGs to transcription factor binding sites and KEGG pathways; (**d**) Supervised feature selection using SKAT-O, XGBoost, ElasticNet machine learning to identify a list of candidate epigenetically-driven differential expressed genes in NO ID JIA patients; (**e**) Differential expression analysis on candidate genes selected by machine learning. (**f**) Interrogating Connectivity MAP (CMAP) tool for identification of potential therapeutic targets. (g) Signature validation on independent datasets *Figure created with biorender.com*

In Fig. 1, we described the overall workflow. After DNAm data preprocessing, we identified stochastic epigenetic mutations (SEMs) and calculated the epigenetic mutation load (EML, natural logarithm of the total number of SEMs) and 19 epigenetic clocks for each patient at the two time points. Then, we conducted linear regression analyses to investigate differences in epigenetic biomarkers between ID and NO ID patients (Fig. 1a and b). The results of the regression analyses indicated EML as the most robust and statistically significant epigenetic biomarker for distinguishing between the two study groups. We annotated epimutated CpG sites on transcription factor binding sites (TFBSs) (Moore et al. 2022) and KEGG pathways (Kanehisa et al. 2017) (Fig.1c), and we performed supervised feature selection using (i) Sequence Kernel Association Test - Optimal SKAT-O (Lee et al. 2012), (ii) Elastic Net Regularized Regression (Zou and Hastie 2005)and (iii) eXtreme Gradient Boosting (XGBoost) (Chen 2016) (Fig.1d). Considering the low samples size, to reduce the risk of type II errors associated with multiple testing correction, and to enhance biological relevance, we preferred a machine learning-based pipeline over classical regression approaches, prioritizing features consistently identified across three independent algorithms, as these were considered more robust and biologically meaningful. Genes participating in multiple significant KEGG pathways or associated with several significant TFBSs were designated as hub genes and subsequently filtered based on differential expression analysis between the two groups (Fig. 1e). This process yielded a final list of genes where the higher number of epimutations in NO ID patients corresponded to differential gene expression (named epigenetically driven up- and down-regulated genes in NO ID patients). Subsequently, the list of candidate up- and down-regulated genes was used as the input for the Connectivity Map (CMAP) tool (Lamb et al. 2006) (Fig.1f), which works by comparing gene expression signatures from association studies to a large database of gene expression profiles produced by various compounds in different cellular lines. The approach is based on the assumption that if a drug signature shows the opposite pattern to a disease signature, that drug can be a potential therapeutic candidate for that disease. Finally, we validated our findings using three independent datasets: (1) genome-wide DNAm profiles from CD4⁺ T cells of 56 JIA patients and 57 age- and sex-matched healthy controls, generated using the Illumina HumanMethylation450 BeadChip; (2) gene expression data from peripheral blood mononuclear cells (PBMCs) of 17 children with rheumatoid factor–negative (RF−) polyarticular JIA, profiled using Illumina HumanWG-6 v3.0 expression BeadChip at two time points: baseline (all patients with inactive disease) and follow-up (8 with active disease, 9 with continued inactive disease); RNA sequencing data from PBMCs of 85 JIA patients spanning three clinical categories: inactive treated, active treated, and active untreated (Fig. 1g).

### Computation of epigenetic clocks

A set of 19 epigenetic clocks, including both first- and next-generation clocks, was computed for all JIA patients to investigate their association with disease clinical activity. Analytical methods for epigenetic clocks calculations are described in the Supplementary Materials.

### Detection of SEMs and computation of EML

Considering DNAm profiles of ID JIA patients at T_0_ as the reference, we computed quartiles and interquartile range (IQR) of methylation values for each CpG probe. Then, we identified SEMs as the extreme values exceeding three times the interquartile range: beta values lower than Q1-(3 x IQR) or higher than Q3+(3 x IQR) for all patients at both T_0_ and T_end_time points. The EML was defined as the natural logarithm of the total number of SEMs per individual as described in previous studies (Yan et al. 2020).

### Association of clinical activity with epimutation load and epigenetic clocks

The association of JIA clinical activity with epigenetic clocks and EML was evaluated using linear regression models, adjusted for age and sex. We tested the association of each epigenetic biomarker with clinical activity at both T_0_ and T_end_ separately. In each model, the epigenetic biomarker was treated as the dependent variable, with clinical activity as the predictor. Effect sizes were expressed as standard deviation differences across all epigenetic biomarkers, enabling the comparison of measures that were originally reported in different units.

### Machine learning feature selection

The first step in the feature selection process involved annotating CpG sites on TFBS using ChIP-Seq data generated from CD4^+^ T cells within the ENCODE project, via the *MeinteR *R package (Malousi et al. 2019), and associating them with KEGG pathways using the *KEGGREST* R package. This resulted in aggregated data, where for each sample, we obtained the number of epimutations associated with 159 TFBS and 358 KEGG pathways.

We then applied three different machine learning approaches (SKAT-O, XGBoost, and Elastic Net Regression) to identify the most relevant TFBSs and KEGG pathways that exhibited a higher number of SEMs in NO ID patients at T_end_. To verify the expression of selected TFs in CD4^+^ T cells, we queried the GSE107011 dataset, which contains 114 RNA-seq samples of sorted immune cell populations, using the R package *celldex*(Aran et al. 2019).

The SKAT-O (Sequence Kernel Association Test - Optimal) procedure is a statistical method originally developed to test associations between rare genetic variants and phenotypes. In this study, we adapted it for rare epigenetic mutations, assuming similar underlying biological mechanisms, where the accumulation of mutations or epimutations leads to altered gene regulation and, potentially, disease progression. SKAT-O combines the strengths of two approaches: the burden test, which aggregates the effects of all variants, and SKAT, which accounts for individual variant effects. The procedure is detailed in Lee et al. (Lee et al. 2012) and is implemented in the R package *SKAT.*

For Elastic Net Regression and eXtreme Gradient Boosting (XGBoost), we used the *LogisticRegression* class from the *linear_model* Python module of scikit-learn (*sklearn*) (Pedregosa 2018) and the *XGBClassifier* from the Python *xgboost *library (Chen 2016), respectively.

Before model training, features were scaled to have average 0 and standard deviation 1, using standard normalization (*StandardScaler* from *sklearn.preprocessing*). Both models were trained using a stratified 5-fold cross-validation (*StratifiedKFold* from Python *sklearn.model_selection*), ensuring class balance within each fold. *GridSearchCV* was used for hyperparameter tuning, with the “average_precision” metric to select the best-performing model based on predefined parameter grids. Further details regarding hyperparameter tuning are described in the Supplementary Materials.

Feature importance for ElasticNet was derived from the absolute values of the model’s coefficients, with non-zero coefficients indicating significant features, while in XGBoost was determined using the ‘weight’ metric, reflecting how frequently a feature was used to split a node across trees.

All the statistical analyses have been performed with R v4.3.3 and Python v3.12.4.

### External validation of epigenetic signature

To validate the epigenetic component of our findings, we analyzed an independent DNAm dataset from a previously published study by Chávez-Valencia et al. (Chavez-Valencia et al. 2018). This dataset includes genome-wide DNAm profiles from CD4⁺ T cells of 56 JIA patients and 57 age- and sex-matched healthy controls, generated using the Illumina HumanMethylation450 BeadChip.

To ensure comparability between discovery and validation datasets, batch effects arising from differences between the two recruitment centers were corrected using the ComBat algorithm (Johnson et al. 2007) as implemented in the *sva*R package (Leek et al. 2012). This empirical Bayes method adjusts for systematic technical variation while preserving biological variation of interest, enabling valid cross-dataset comparisons.

Identification of SEMs, computation of the EML, and annotation of epimutated CpG sites on the ComBat-corrected dataset were performed using the same analytical pipeline used in the discovery dataset. SEMs were then annotated and tested for enrichment within the genes and TFBSs prioritized in the discovery phase using SKAT-O. Pathway enrichment analysis on the list of significant genes was performed using the Molecular Signature Database (MSigDB) (Liberzon et al. 2015) database via the*enrichR *R package (Kuleshov et al. 2016).

### External validation of transcriptomic signature

To validate the transcriptomic signature related findings, we downloaded two independent datasets from the GEO repository (GSE26112 and GSE79970), both of them including transcriptomic data from JIA patients. Unlike our primary dataset, these validation datasets do not include DNAm data and were generated from peripheral blood mononuclear cells (PBMCs) rather than purified CD4^+^ T cells. GSE26112 included gene expression profiles from 17 children with rheumatoid factor negative (RF-) polyarticular JIA, measured using Illumina HumanWG-6 v3.0 expression BeadChip. Samples were collected at two time points: baseline (all patients with inactive disease) and follow-up (8 patients with active disease, 9 with continued inactive disease).

GSE79970 consisted of RNA sequencing data from PBMC of 85 JIA patients across three clinical categories: inactive treated patients, active treated patients, and active untreated patients. For each dataset, we performed differential expression analyses using *DESeq2 *R package (Love et al. 2014) comparing inactive vs. active JIA patients (adjusting for treatment in the GSE79970 dataset) and combined the results through random effect meta-analysis using the*metafor *R package (Viechtbauer 2010). Differentially expressed genes with consistent direction of fold change compared to our signature have been cross-referenced with the MSigDB to investigate possible enrichment with JIA- and inflammation-related pathways.

## Results

### Significant association between EML and clinical activity

**Fig. 2:**
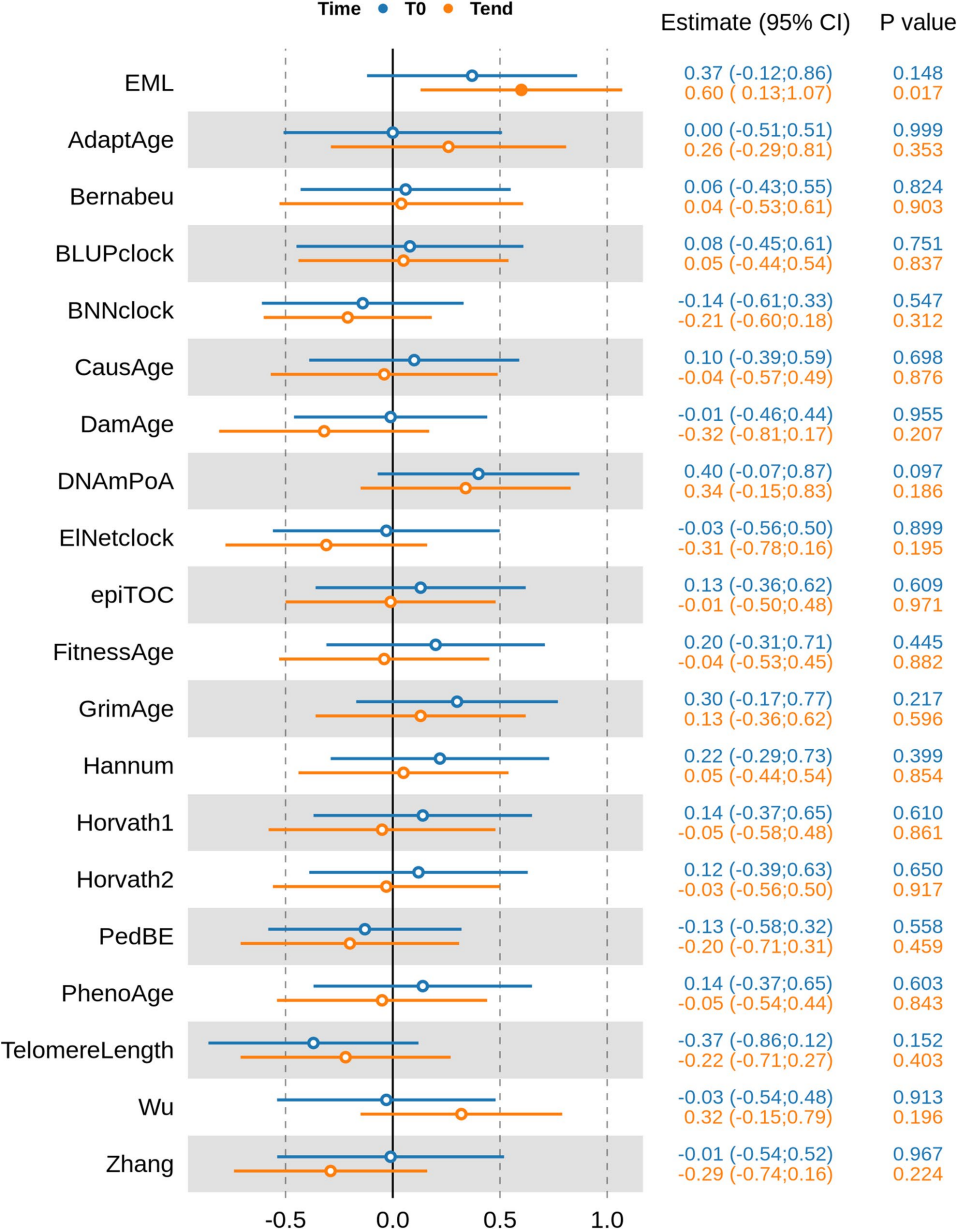
**–** Results from the linear regression analyses presented as a forest plot. Estimates (with 95% confidence intervals) for the differences between ID and NO ID patients across various epigenetic clocks are presented at two time points, T_0_ (blue) and T_end_ (orange). EML values are higher in NO ID compared to ID patients at both T_0_ and T_end_, with a statistically significant difference observed at T_end_ (β = 0.60; 95% CI [0.13–1.07]; *p* = 0.02). Instead, no epigenetic clock has been found to be associated with JIA clinical activity

We investigated differences in EML and 19 epigenetic clocks by clinical status (ID vs. NO ID), through linear regression analyses, at T_0_ and at T_end_, respectively, using the epigenetic biomarkers as dependent variables and the disease activity as the predictor adjusting for age and sex. The overall results are summarized in Fig. 2. Effect sizes were standardized (expressed as standard deviation differences between the two groups) to enable comparison across different epigenetic biomarkers, which are originally expressed in varying units of measurement. We observed higher EML values in NO ID compared to ID patients at T_0_ (β = 0.37; 95% CI [−0.12–0.86]; *p* = 0.15), with this difference becoming more pronounced and statistically significant at T_end_ (β = 0.60; 95% CI [0.13–1.07]; *p* = 0.02).

### Multi-omic feature selection identified candidate genes

**Fig. 3:**
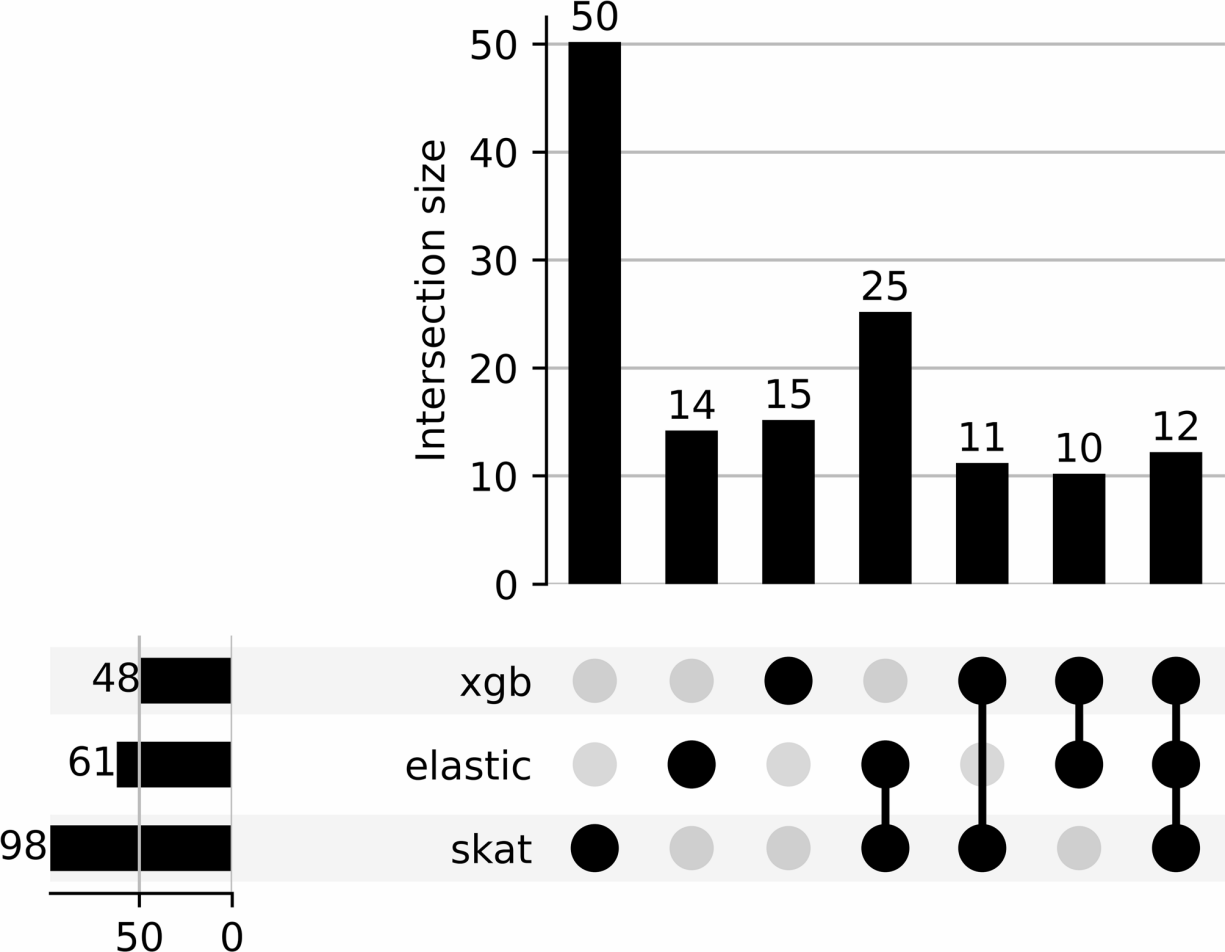
**-** The upSet plot showing the overlap of TFBSs selected by three different methods: XGBoost (xgb), ElasticNet (elastic), and SKAT-O (skat). Bars in the top panels represent the number of intersecting features for each combination of methods, while the bars on the left panels indicate the total number of features selected by each method independently. Black dots connected by lines indicate the specific combinations of methods contributing to the intersection size

Based on the above findings, we focused our investigation on EML. Results from the linear regression analyses indicate a higher number of epimutated CpG sites in NO ID than ID patients across the whole genome. To prioritize the genes showing the most relevant differences between ID and NO ID patients, we employed three distinct feature selection approaches using SKAT-O, Elastic Net Penalized regression, and XGBoost. We considered the features selected by all the three approaches as the most robust candidates, deserving additional investigation for identifying potential biomolecular pathways involved in diseases reactivation and potential therapeutic targets. For this analytical step, we focused exclusively on the epigenetic and transcriptomic data at T_end_. This allowed us to concentrate on genes exhibiting increased epimutations likely driven by disease reactivation, which appears to be the predominant mechanism according to the linear regression results. The available sample size did not allow a gene-level comparison between the two groups with enough statistical power. Indeed, we conducted SKAT-O, ElasticNet, and XGBoost analyses, aggregating data at the gene level. However, this level of aggregation did not sufficiently capture significant differences between the two groups in the SKAT-O analysis, nor did it yield robust classification results with ElasticNet and XGBoost (data not shown). Therefore, we annotated the epimutated CpG sites into TFBSs according to the ChipSeq experiments performed by the ENCODE consortium in CD4^+^T cells (Moore et al. 2022), and into KEGG pathways (Kanehisa et al. 2017). This approach allowed to aggregate epimutated CpG sites within 159 TFBS and 358 KEGG pathways reducing the number of features and consequently increasing the statistical power. Using the described CpGs aggregations, SKAT-O identified 98 TFBSs that showed a significantly higher number of SEMs in NO ID than ID with false discovery rate (FDR) adjusted p-value lower than 0.1. No KEGG pathways reached statistical significance at this threshold. Results are presented in Supplementary Table S1 and Table S2. Elastic Net and XGBoost classification algorithms were trained using a 5-fold cross-validation framework, with hyperparameters optimization through grid search. Feature selection was performed by evaluating the importance metrics unique to each model: non-zero coefficients and the frequency of their use in tree splits, for ElasticNet and XGBoost, respectively. Detailed performance metrics for XGBoost and ElasticNet models, including F1 score, accuracy, precision, recall, and average precision, are summarized in Supplementary Table S3. Using the described procedure, ElasticNet identified 61 TFBSs and 50 KEGG pathways (Supplementary Table S4 and Table S5), while XGBoost identified 48 TFBSs and 84 KEGG pathways (Supplementary Table S6 and Table S7). Considering the overlap among the three feature selection approaches, we selected 12 TFBSs (Fig. 3) as the most relevant in the comparison of ID with NO ID JIA patients. According to GSE107011 dataset, 10 out of the 12 TFs targeting the selected TFBSs were indeed expressed in CD4^+^ T cells (Supplementary Table S8). No KEGG pathway was identified by all 3 machine learning models. The list of selected TFBSs was further analyzed to identify hub genes which were defined as those mapping to multiple TFBSs. In Supplementary Table S9, we List the top 100 genes associated with the 12 TFBSs identified by machine learning, ranked by their frequency in each annotation. By using the frequency of genes annotated to multiple TFBSs, we observed a robust selection of hub genes with several of them being regulated by more than 80% of the 12 selected TFBSs and the gene *FOXP1* targeted by all 12. Based on the above, we defined the List of candidate genes as those that are targeted by at least 50% of the identified TFs (*N* = 909). As the last step, we further prioritized genes in which a higher number of SEMs in NO ID than ID led to a significant difference in gene expression profile. For this step, we performed a differential gene expression analysis using transcriptomic data from the 909 candidate genes at T_end_. We found 157 significant genes (FDR < 0.2), of which 80 were up- and 77 down-regulated in NO ID (Supplementary Table S10, and Figure S4).

### Connectivity MAP identified potentially clinically actionable drugs

As the last step, we used the CMAP tool to identify a list of potential drugs that counteract the identified signatures associated with clinical reactivation of JIA, and that could potentially be used in the clinical practice (Lamb et al. 2006). Previously selected genes were used as input for CMAP and it provided a list of candidate compounds, ranked by the raw connectivity score which is proportional to their ability to reverse the gene expression signatures associated with the disease reactivation. We only considered chemical compound drugs statistically significant after FDR correction for multiple testing (q < 0.05) and a negative normalized connectivity score. We focused on a preselected set of cell lines suitable to investigate the effect of a drug in the context of an autoimmune disease like JIA. The selected cell lines include: ‘*JURKAT*’, ‘*THP1*’, ‘*K562*’, ‘*HL60*’, ‘*BJAB*’, ‘*NALM6*’, ‘*CD34*’ and their full description is provided in Supplementary Table S11. According to the described procedure, we identified a List of 12 candidate compounds that met al.l the filtered criteria, listed in Table 2.

**Table 2:** – Results from the CMap tool showing potential therapeutic targets for JIA. *Cell name*: cell line in which the perturbation was tested. *Compound id*: BRD ID, or broad ID, is an identifier used to uniquely address a particular small molecule. It was developed by the broad Institute and is used throughout CMap data to identify chemical perturbagens. *Compound name*: internal (CMap-designated) name of a perturbagen. By convention, for genetic perturbations CMap uses the HUGO gene symbol. *Compound dose*: concentration at which the perturbation was applied, reported in micromolar (uM). *Compound time*: duration of exposure (in hours) of cells to the perturbagen. *# genes in the signature*: signature strength is a measure of the magnitude of the response elicited by a given treatment and is computed as the number of landmark genes (out of 978) with absolute z-score greater than or equal to 2. *Transcriptional activity*: perturbagen’s transcriptional activity. The more transcriptionally active a perturbagen is, the higher its transcriptional activity. *Mechanism of action*: describes the known Pharmacological activity of each compound. *Target name*: target genes or proteins modulated by the compound. *Raw connectivity score*: represents the correlation between the input gene expression signature and the compound’s gene expression profile. *Normalized connectivity score*: computed by dividing the Raw connectivity scores by the signed-mean scores of signatures. *FDR q*: false discovery rate adjusted p-value of the CMap enrichment analysis

Cell name	Compound id	Compound name	Compound dose	Compound time	# of genes in the signature	Compound transcriptional activity	Mechanism of action	Target name	Raw connectivity score	Normalized connectivity score	FDR q
JURKAT	BRD-A33746814	avicin-g	0.37 μm	24 h	482	0.5	NFKB inhibitor	NaN	−0.4	−1.51	4.47 × 10^−16^
THP1	BRD-K12683773	methyl-fasudil	10 μm	6 h	252	0.4	NaN	NaN	−0.43	−1.65	2.24 × 10^−16^
BJAB	BRD-K03390685	cobimetinib	10 μm	4 h	214	0.33	MEK inhibitor	NaN	−0.4	−1.52	2.24 × 10^−16^
THP1	BRD-K32330832	VER-155,008	12 μm	6 h	86	0.12	HSP inhibitor	HSPA1A	−0.38	−1.44	0.0339
JURKAT	BRD-A49838158	phenprocoumon	0.03 μm	24 h	77	0.1	Vitamin K antagonist	VKORC1	−0.38	−1.45	0.0302
K562	BRD-K32972437	BRD-K32972437	10 μm	4 h	76	0.12	NaN	NaN	−0.38	−1.45	0.0302
NALM6	BRD-K20285085	fostamatinib	10 μm	24 h	275	0.37	Syk inhibitor	NaN	−0.39	−1.46	0.0195
THP1	BRD-K65170927	aprepitant	0.37 μm	24 h	195	0.11	Tachykinin antagonist	TACR1|CYP2C19	−0.39	−1.47	0.0186
JURKAT	BRD-K42495768	tasisulam	0.01 μm	24 h	126	0.18	Apoptosis stimulant	NaN	−0.39	−1.47	0.0151
JURKAT	BRD-A45498368	WYE-125,132	1.11 μm	24 h	568	1.08	MTOR inhibitor	MTOR|PIK3CA	−0.39	−1.5	0.0041
THP1	BRD-K68392338	ZK-93,426	0.04 μm	24 h	96	0.11	Benzodiazepine receptor antagonist	GABRA1|GABRA2|GABRA3|GABRA5	−0.4	−1.5	0.0019
THP1	BRD-K12423485	griseofulvin	10 μm	24 h	220	0.18	Tubulin inhibitor	KRT12	−0.4	−1.5	0.0014

### External validation confirms higher EML in active JIA patients

Application of the ComBat algorithm successfully harmonized the DNAm data distributions between discovery and validation datasets (Supplementary Figure S5).

Linear regression analysis adjusted for age and sex showed significantly higher EML levels in active JIA patients from the validation dataset compared to inactive JIA patients from the discovery set (*p* = 0.04), although the effect size was smaller than that observed when comparing NO ID and ID patients in the discovery dataset. In contrast, no significant differences were found between healthy controls from the validation dataset and inactive JIA patients from the discovery group (Supplementary Figure S6).

Furthermore, SKAT-O analysis revealed a significantly higher SEM burden in active JIA patients compared to healthy controls in 7 out of 12 TFBSs and in 16 out of 157 genes prioritized in the discovery set (Supplementary Tables S12, S13). The direction and magnitude of the SEM burden across the 12 TFBSs were overall confirmed in the validation cohort, with a positive correlation in log₂FC values (ρ = 0.46; Figure S7).

Pathway enrichment analysis of genes showing higher SEM burden in JIA patients than healthy controls revealed enrichment in key inflammatory pathways, including ‘*TNF-alpha signaling via NF-kB’* and ‘*TGF-beta signaling’*, which has been previously implicated in JIA and rheumatoid arthritis (Zhang et al. 2023; Singh et al. 2012; Bira et al. 2005), further supporting the biological relevance of our findings (Supplementary Table S14).

### External validation refined JIA transcriptomic signature revealing candidate inflammation pathways

Differential gene expression analyses on independent external datasets were performed comparing inactive vs. active JIA patients and combined the results through meta-analysis. Among the genes in our signature, 22 showed significant differential expression (FDR-adjusted p-value < 0.2) with consistent direction of fold change compared to our original analysis (15 up-regulated; 7 down-regulated genes, see Supplementary Table S15). Gene set enrichment analysis on the reduced gene list against the MSigDB revealed significant enrichment in inflammatory and immune related pathways like ‘*TNF-alpha signaling via NF-kB*’, ‘*KRAS signaling*’, and ‘*TGF-beta signaling*’ (see Supplementary Table S16), consistent with results of the enrichment analysis on differentially epimutated genes, demonstrating convergent evidence between epigenetic and transcriptomic signatures across different validation datasets.

## Discussion

We re-analyzed a publicly available dataset comprising DNAm and gene expression data from 44 JIA patients. Samples were collected at two key time points: upon withdrawal of anti-TNF therapy (T_0_) and eight months later, during which 14 out of 44 patients experienced disease reactivation (T_end_). For our analysis, we focused on comparing patients who maintained inactive disease with those who did not, excluding 24 patients who had acute flares at T_end_. The decision to exclude “flares” patients from the analysis follows the observation in the original study by Spreafico et al. (Spreafico et al. 2016), which found no significant differences in DNAm or transcriptomic profiles between ID and flare patients, while both groups differed markedly from the intermediate NO ID group. Spreafico and colleagues hypothesized that during acute flare events, pathogenic immune cells rapidly extravasate to peripheral tissues, leaving minimal and transient epigenetic changes in circulating cells, thereby making the DNAm profiles of flare patients more similar to those of ID patients than to NO ID patients, who exhibit sustained immune dysregulation. Based on this rationale, we chose to focus our comparison on ID versus NO ID patients, accepting a reduction in statistical power to avoid overlooking relevant biomarkers potentially masked by the inclusion of flare patients. Nevertheless, our sensitivity analyses demonstrated that including “flares” patients yielded consistent results. In the original study (Spreafico et al. 2016), the authors employed weighted gene co-expression network analysis (WGCNA) (Langfelder and Horvath 2008) to identify biologically coherent modules and investigate their associations with clinical disease activity and other relevant features. Their findings highlighted a potential role of epigenetic variability within the major histocompatibility complex (MHC) in driving disease activation. Furthermore, they proposed that epigenetic profiles could distinguish between different states of clinical activity and identified T-cell–related biological functions as being strongly linked to, and possibly predictive of, clinical outcomes.

### Epigenetic mutation load as a biomarker of disease reactivation

We adopted a different analytical approach, focusing on rare stochastic epigenetic mutations (SEMs) and epigenetic clocks as potential biomarkers for predicting JIA reactivation. This strategy was guided by recent literature suggesting a significant role of SEMs in autoimmune diseases, including adult rheumatoid arthritis (Yan et al. 2020; Gao 2012; Surace and Hedrich 2019). We observed higher epigenetic mutation load (EML) in JIA patients with disease reactivation (NO ID) compared to those maintaining inactive disease (ID) at both T_0_ and T_end_, with a statistically significant difference observed at T_end_. These results suggest that elevated EML could both contribute to and result from disease reactivation, highlighting its potential as a predictive biomarker for monitoring disease status. The differences between the two groups were more pronounced at T_end_, leading us to interpret the EML increase as likely a consequence of JIA clinical reactivation.

Although some previous studies have examined SEM burden in pediatric populations (Spada et al. 2020), to our knowledge, none have investigated its longitudinal trajectory to estimate the rate of accumulation under physiological conditions. This gap limits the ability to contextualize our findings against a reference healthy population. Nonetheless, our results indicate that EML increases over time in JIA patients, with a significantly faster rate observed in NO ID compared to ID. Worthy of note, the presence of slightly higher EML in NO ID compared to ID patients at T_0_ (when all patients have no clinical symptoms), despite not statistically significant, suggests that epigenetic changes might precede clinical symptoms. If confirmed in future studies, this would have important implications for early disease monitoring and a deeper understanding of disease etiology. Instead, we found no evidence of association between epigenetic clocks and JIA clinical activity.

### Machine learning-driven gene prioritization

Based on the above described findings, we performed additional machine learning-based analyses aiming to identify the most relevant genes exhibiting a higher EML in NO ID compared to ID patients. Given the Limited statistical power in a gene-level genome-wide scan of 14 vs. 30 patients, we instead annotated CpGs based on the TFs that target them and the KEGG pathways they pertain to. The use of three distinct feature selection methods for TFBSs and KEGG pathways prioritization significantly enhances the robustness of our findings. We focused on features extracted by all three approaches to reduce false positive rate and increase the reliability of the selected biomarkers. Importantly, all three methods effectively addressed the class imbalance between ID and NO ID groups, avoiding possible bias in the results. Furthermore, hyperparameters for XGBoost and ElasticNet were properly tuned to mitigate overfitting issues related to the Limited sample size. Our results show that the three algorithms demonstrated greater concordance in feature ranking when CpGs were annotated by TFBSs compared to grouping by KEGG pathways. Specifically, 12 different TF proteins were selected by all three machine learning methods, 10 of which are expressed in CD4^+^T cells according with reference dataset (Xu et al. 2019).

A crucial step in our gene prioritization process was the integration of epigenetic and transcriptomic data, enabling us to definitively prioritize genes where variations in the total number of SEMs corresponded to significant changes in gene expression. This multi-omic approach strengthens the biological relevance of the identified up- and down-regulated genes, allowing us to identify an epigenetically-driven gene expression signature of JIA clinical activity.

### Exploratory clinical insights from CMAP

The derived signature was used as the input for the Connectivity Map (CMAP) tool (Chen 2016) which compares a user-provided gene List of up-regulated and down-regulated genes with a database of gene expression profiles from various cell types treated with different chemical compounds and genetic perturbations to identify potential drugs or compounds that could reverse the gene expression signature of interest. The CMAP analysis identified 12 compounds that significantly counteracted the transcriptomic signature. Among the list of candidate compounds identified by CMAP, several have already shown potential clinical utility. The most significant association was found for the*avicin-g* (BRD-A33746814), a *nuclear factor kappa B (NF-kB)* inhibitor. The *NF-kB signaling pathway*plays a key role in JIA by regulating the expression of pro-inflammatory genes (Zhang et al. 2023). Another significant compound is *methyl-fasudil* (BRD-K12683773), a derivative of *fasudil*, a well-known Rho-kinase inhibitor. Although *fasudil* is primarly used for treating cardiovascular conditions, evidence suggests it also modulates *NF-kB signaling *and shows beneficial effects on rats with RA (Okamoto et al. 2010). *Cobimetinib* (BRD-K03390685) is *MEK* inhibitor. Although *MEK *inhibitors are mainly used for cancer treatment, they have been shown to be candidate agents for the treatment of systemic JIA (Zhang et al. 2021). Among the other compounds, fostamatinib is a *SYK inhibitor *involved in B-cell and innate immune cell activation, and has been tested in RA clinical trials with positive outcomes (Tanaka et al. 2021). Additionally, *aprepitant *(BRD-K65170927) has shown anti-inflammatory effects in RA models (Pavithra et al. 2024; Liu et al. 2019).

### External validation

External validation of our findings in an independent set of Australian JIA patients enrolled at the Royal Children’s Hospital in Melbourne supports the reliability of our results. Active JIA patients demonstrated significantly higher EML compared to healthy controls, consistent with findings in the discovery set. Furthermore, SKAT-O analysis of SEMs within candidate TFs validated 7 out of 12 identified targets, providing robust independent confirmation of our epigenetic signature. Pathway enrichment analysis of differentially epimutated genes revealed significant overrepresentation of key inflammatory pathways, including *TNF-alpha signaling via NF-κB* and *TGF-beta signaling *which has been previously implicated in JIA and rheumatoid arthritis (Zhang et al. 2023; Singh et al. 2012; Bira et al. 2005).

For validating the epigenetically driven transcriptomic signature, we leveraged gene expression profiles from PBMCs in two independent datasets comparing active versus inactive JIA patients. Despite cross-tissue Limitations, 22 genes from our signature showed significant differential expression with consistent directional changes relative to our CD4^+^ T cell findings. Enrichment analysis of these validated genes identified significant overrepresentation of inflammatory pathways, notably including *TNF-alpha signaling via NF-κB* and *TGF-beta signaling*, showing consistent results across different omics and datasets.

This remarkable convergence of pathway-level findings across both epigenetic and transcriptomic validation, derived from independent cohorts and complementary molecular approaches, strongly supports the biological robustness of our results and highlights these inflammatory cascades as promising therapeutic targets for JIA.

### Strengths and limitations

We acknowledge some Limitations in this study. The sample size in the discovery dataset is small for whole-genome DNAm and transcriptomic analyses, with only 14 patients in the NO ID class. This is primarily due to the rarity of the disease. However, we employed a hypothesis-driven approach, guided by preliminary observations of higher epimutation load in NO ID compared to ID patients, rather than an agnostic investigation based on classical linear regression analyses which have a high risk of type II errors due to multiple testing correction. Furthermore, a key strength of the discovery dataset is the availability of both DNAm and transcriptomic measurements at two time points, allowing for longitudinal analyses.

The limited sample size may also pose challenges for machine learning analyses and feature prioritization. To address this, we adopted an analytical strategy involving three different machine learning methods and focused on results consistently identified across all three. This approach was intended to reduce the type I error rate and enhance the robustness of our findings. Moreover, external validation of the results related to the prioritized TFs in an independent dataset highlights the reproducibility of our findings.

We did not apply multiple testing correction when identifying differentially expressed genes in the discovery set. However, this analysis was based on a candidate gene list derived from the DNAm machine learning analysis. The exploratory nature of subsequent steps supports this decision but warrants cautious interpretation of the findings, despite alignment with previous literature that increases our confidence about this study’s findings. We also acknowledge that some differentially expressed genes showed modest fold changes, which may increase the likelihood of false positives. Nevertheless, key results were replicated in two independent transcriptomic datasets, and consistent enrichment of relevant immune-related pathways across both methylation and expression data increases our confidence in the biological relevance of the findings.

One strength of this study is the reanalysis of a publicly available dataset using updated analytical methods, which offers new insights into the biomolecular mechanisms of JIA clinical activity. Additionally, we introduced a novel multi-omic approach by characterizing epimutated CpGs and annotating them to transcription factor binding sites. This method can be potentially applied in the context of other epigenetically dysregulated diseases to identify epigenetically-driven alterations in gene expression. Furthermore, external validation of our transcriptomic signature on two independent datasets showed encouraging results, and enriched molecular pathways were consistent with those identified from differentially epimutated genes. While immediate clinical translation is beyond the scope of this work, we believe that this integrative approach has the potential to inform future translational advances. Specifically, targeting the *NF-κB signaling* pathway appears promising and warrants further investigation. Finally, we acknowledge no functional validation was performed. However, the convergence of evidence from validation epigenetic and transcriptomic datasets provides strong support for the biological relevance of our findings.

## Conclusions

This study highlights a significant association of epigenetic mutations with JIA clinical activity, suggesting a critical role of epigenetic mutations in the reactivation of JIA. We describe molecular mechanisms potentially implicated in JIA etiology and clinical reactivation of the disease. Future studies are required to accumulate epidemiological evidence and to assess the efficacy of the identified compounds.

Further, this study presents a novel multi-omic methodological approach to identify epigenetically-driven gene expression modifications moving beyond traditional EWAS approaches, potentially useful to investigate other multifactorial diseases. Our approach integrates the annotation of epimutated CpG sites to the TFs they are targeted, providing a more comprehensive understanding of the molecular mechanisms underlying disease.

## Supplementary Information

Supplementary Material 1.

Supplementary Material 2.

Supplementary Material 3.

Supplementary Material 4.

Supplementary Material 5.

Supplementary Material 6.

Supplementary Material 7.

Supplementary Material 8.

Supplementary Material 9.

Supplementary Material 10.

## Data Availability

No datasets were generated or analysed during the current study.

## Abbreviations

JIA: Juvenile idiopathic arthritis
ID: Inactive disease
NO ID: Not inactive disease
DNAm: DNA methylation
TNF: Tumor necrosis factor
EWAS: epigenome-wide association study
SEM: Stochastic epigenetic mutation
EML: Epigenetic mutation load
RA: rheumatoid arthritis
UMAP: uniform manifold approximation and projection
TFBS: Transcription factor binding sites
TF: Transcription factor
SKAT-O: Sequence Kernel Association Test – Optimal
XGBoost: eXtreme Gradient Boosting
CMAP: Connectivity Map
FDR: false discovery rate
MHC: major histocompatibility complex
sJIA: systemic JIA
PRC2: Polycomb Repressive Complex 2
HDAC: histone deacetylase
VEGFR: vascular endothelial growth factor receptor
DMARD: Disease-modifying antirheumatic drug
IQR: interquartile range
BNN: Bayesian Neural Network
PedBE: pediatric buccal cells
BLUP: Best Linear Unbiased Prediction
AA: age acceleration
WBC: White blood cell
