# Supplementary figures and images for: Stochastic epigenetic mutation profiles as biomarkers of clinical activity in juvenile idiopathic arthritis: a multi-omic machine learning approach for gene prioritization
Source: Mol Med. 2025 Sep 25;31:289. doi: 10.1186/s10020-025-01348-6 (PMC12465343; doi:10.1186/s10020-025-01348-6)

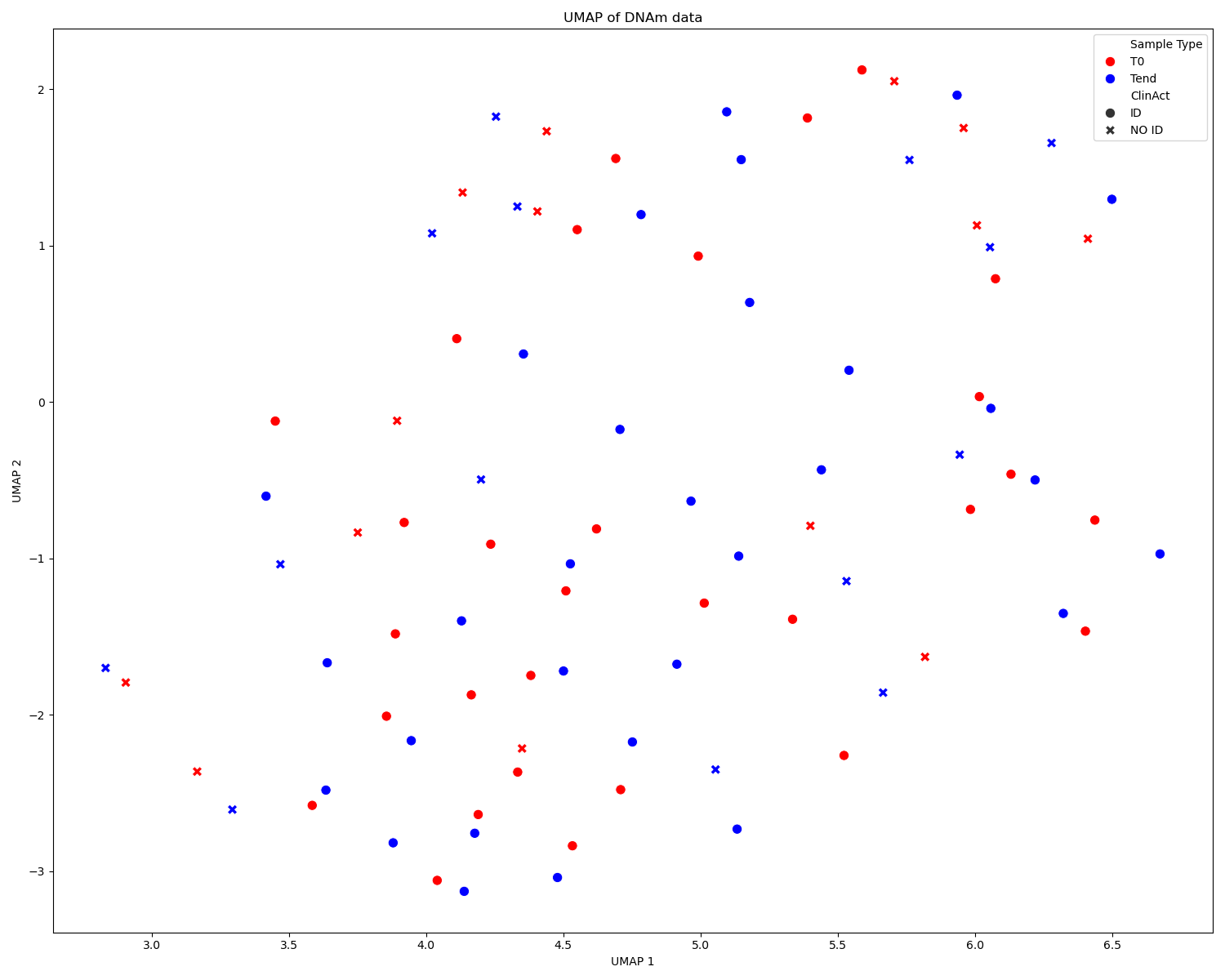

Supplement: Supplementary file 1 — Supplementary Material 1. [file 10020_2025_1348_MOESM1_ESM.png]

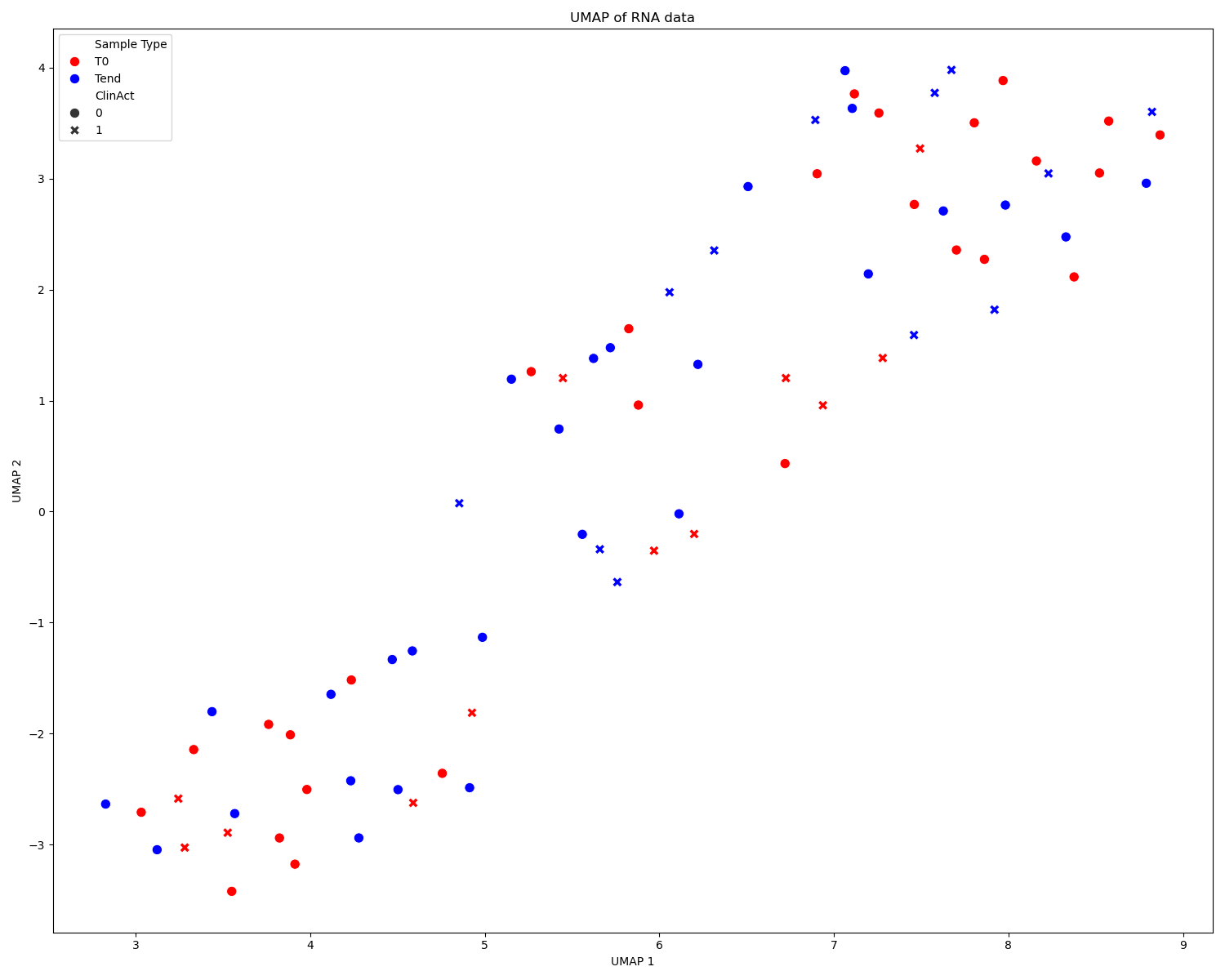

Supplement: Supplementary file 2 — Supplementary Material 2. [file 10020_2025_1348_MOESM2_ESM.png]

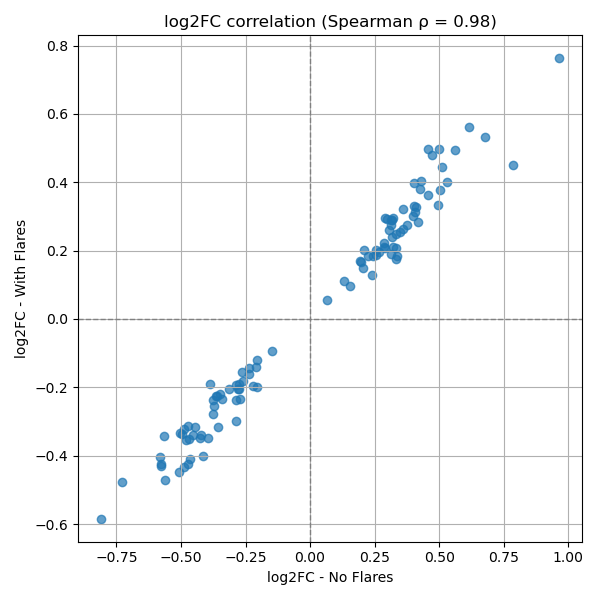

Supplement: Supplementary file 3 — Supplementary Material 3. [file 10020_2025_1348_MOESM3_ESM.png]

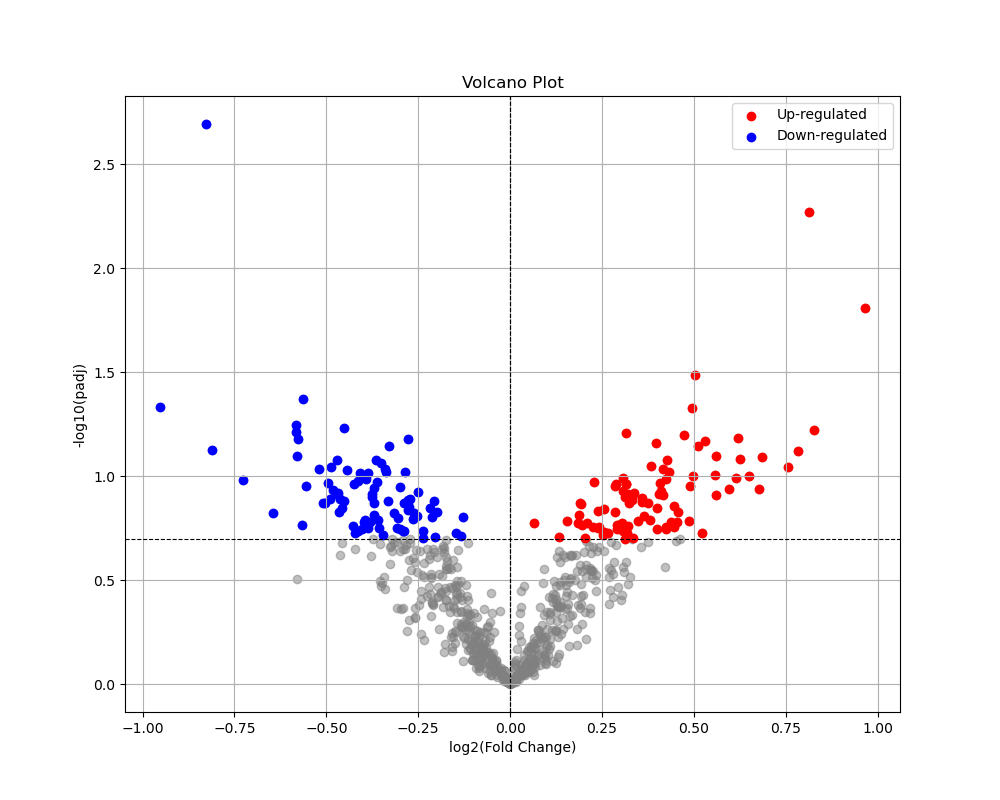

Supplement: Supplementary file 4 — Supplementary Material 4. [file 10020_2025_1348_MOESM4_ESM.png]

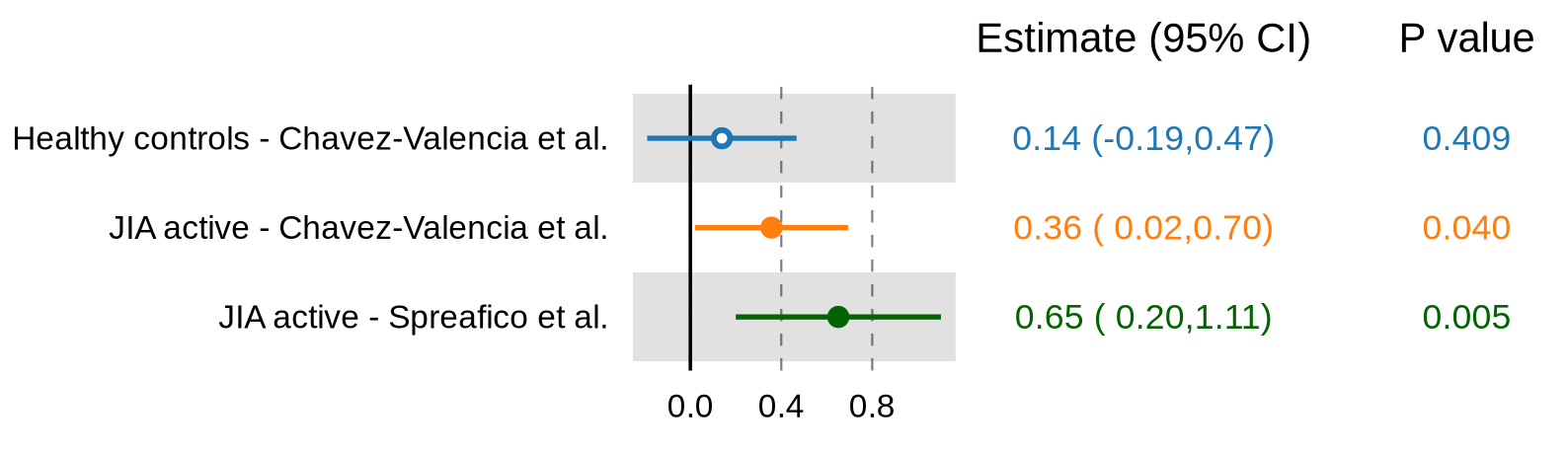

Supplement: Supplementary file 5 — Supplementary Material 5. [file 10020_2025_1348_MOESM5_ESM.png]

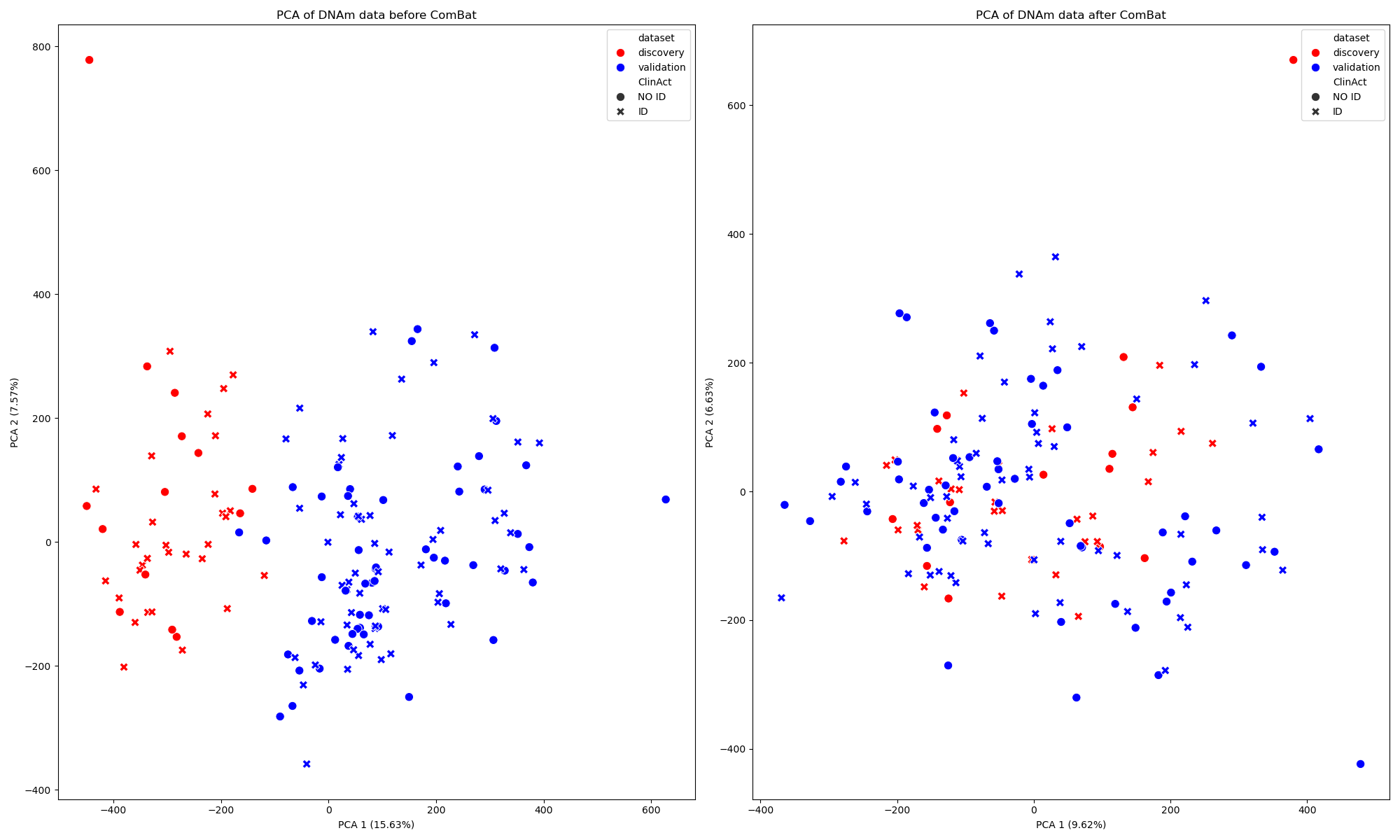

Supplement: Supplementary file 6 — Supplementary Material 6. [file 10020_2025_1348_MOESM6_ESM.png]

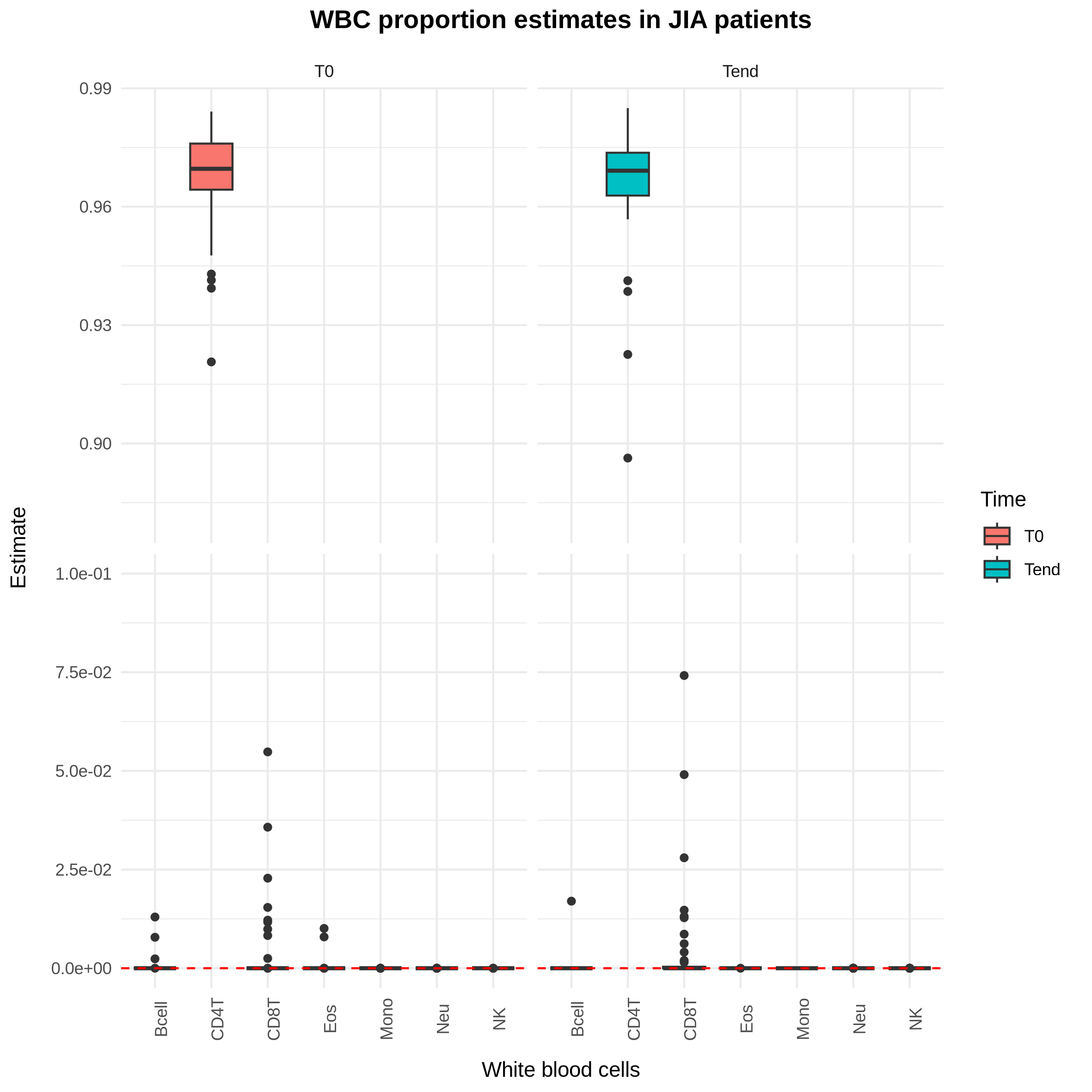

Supplement: Supplementary file 7 — Supplementary Material 7. [file 10020_2025_1348_MOESM7_ESM.png]

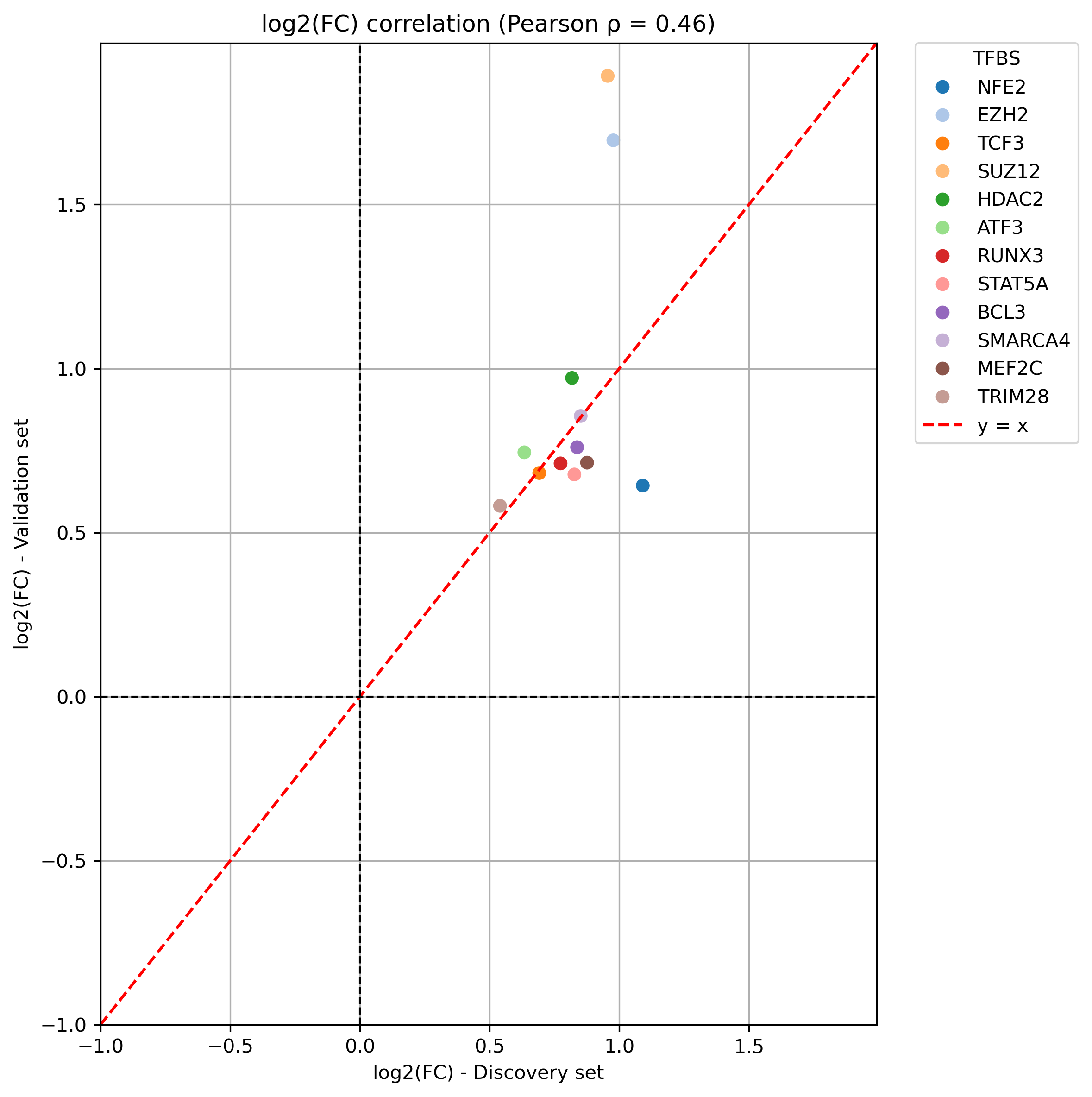

Supplement: Supplementary file 8 — Supplementary Material 8. [file 10020_2025_1348_MOESM8_ESM.png]
