# Supplementary material for: Stochastic epigenetic mutation profiles as biomarkers of clinical activity in juvenile idiopathic arthritis: a multi-omic machine learning approach for gene prioritization
Source: Mol Med. 2025 Sep 25;31:289. doi: 10.1186/s10020-025-01348-6 (PMC12465343; doi:10.1186/s10020-025-01348-6)
Supplement: Supplementary file 10 — Supplementary Material 10. [file 10020_2025_1348_MOESM10_ESM.docx]

**Supplementary Materials for “Stochastic epigenetic mutation profiles as biomarkers of clinical activity in juvenile idiopathic arthritis: a multi-omic machine learning approach for gene prioritization”**

### **Computation of epigenetic clocks**

A set of 19 epigenetic clocks was computed for all JIA patients at the two time points. Briefly, each epigenetic clock is computed as a linear combination of a specific set of CpG sites with varying number of CpGs and weight based on the specific epigenetic clock. We considered the following epigenetic clocks: (i) principal components adjusted version of Horvath original pan tissue clock (Horvath v1), Horvath skin and blood clock (Horvath v2), Hannum, Levine’s DNAmPhenoAge, DNAmGrimAge and DNAmTL as described by Higgins-Chen et al. [1], (ii) Bernabeu and Zhang epigenetic clocks as implemented in Hillary et al. [2], (iii) DNAmAdaptAge, DNAmDamAge and DNAmCausAge epigenetic clocks by Ying et al. [3], (iv) DNAmFitAge epigenetic clock by Jokai et al. [4], the mitotic age tracking of the number of stem-cell divisions proposed by Teschendorff et al. [5], and (vi) epigenetic clocks developed using non-linear analytical approaches like the Bayesian Neural Network (BNN), the pediatric buccal cells (PedBE) clock, Wu’s clock design to predict biological age in children, the Best Linear Unbiased Prediction (BLUP) clock, the Zhang Elastic Net (EN) clock, the predictor of gestational age DNAmGA, as implemented in the *methylclock* R package by Pelegí-Sisó et al. [6]. For each clock, we computed the epigenetic age acceleration (AA) as the residuals of the linear regression of the epigenetic age on chronological age. White blood cell (WBC) proportions were estimated according to the procedure implemented in the *methylclock* R package. The estimated WBC proportions, shown in **Figure S3**, confirm that the DNAm data were derived from CD4+ T cells. As a result, we did not adjust for white blood cell (WBC) proportions in our analyses, although it is a common practice in epigenetic studies.

###

### **Hyperparameter tuning**

We applied three different machine learning approaches: SKAT-O, XGBoost, and Elastic Net Regression.

For SKAT-O we used the R package SKAT. Briefly, SKAT-O combines the output of a classical burden test and a Sequence Kernel Association Test (SKAT) test [7], adaptively selecting the best linear combination of the two association tests. The algorithm computes an empirical p-value by evaluating multiple values of ρ, representing the weight of a given feature. We used a grid search including 15 possible values of ρ equally distributed within the [0,1] interval. Empirical p-values were computed using ‘boostrap’ option in the SKAT function with n.Resampling = 10^06^.

For Elastic Net Regression and eXtreme Gradint Boosting (XGBoost), we used the *LogisticRegression* class from the *linear_model* Python module of scikit-learn (*sklearn*) [8] and the *XGBClassifier* from the Python *xgboost* library [9], respectively.

Before model training, we normalized features to have average 0 and standard deviation 1, using *StandardScaler* from *sklearn.preprocessing*).

To train Elastic Net and XGBoost models, we performed a stratified 5-fold cross-validation using *StratifiedKFold* from Python *sklearn.model_selection*, ensuring class balance within each fold.

*GridSearchCV* from *sklearn.model_selection* was used for hyperparameter tuning, with the “average_precision” metric to select the best-performing model based on predefined parameter grids.

For ElasticNet, the LogisticRegression hyperparameters were tuned as follows: **penalty** was set to "elasticnet" and **l1_ratio** to 0.5, ensuring a balanced combination of L1 and L2 regularizations. The **solver** hyperparameter was specified as "saga" [10], being the only solver compatible with the elasticnet **penalty**. The regularization strength (**C**) was tested at values of 1 (default), 5, and 10. Stopping tolerance (**tol**) was evaluated at 10^-4 and 10^-3, and the maximum number of iterations (**max_iter**) was tested at 5,000 and 10,000 to allow the solver to converge. Finally, **class_weight** was set to "balanced" to adjust the weights to balance the different sample sizes in the two output classes.

Feature importance for ElasticNet was derived from the absolute values of the model's coefficients, with non-zero coefficients indicating significant features.

For XGBoost model, the XGBClassifier hyperparameters were tuned as follows: **n_estimators** was tested with 200 and 500 boosting rounds. The learning rate (**eta**) was tested at 0.3 (default) and 0.1. The L2 regularization term (**lambda**) was validated for 2, 1 and 0 to test regularization at different strengths. The **colsample_bytree** hyperparameter representing the subsample ratio of columns considered by each tree in the model was tested at 0.3, 0.5, and 0.7 rates. Finally, **scale_pos_weight** was computed using the *compute_class_weight* function from *sklearn.utils.class_weight* to handle class imbalance.

Feature importance in XGBoost was determined using the 'weight' metric, reflecting how frequently a feature was used to split a node across trees.

All the statistical analyses have been performed with R v4.3.3 and Python v3.12.4.

**Sensitivity analysis (SA)**

To assess the impact of including the 24 Flare patients excluded from the main analysis, we conducted a sensitivity analysis by aggregating these patients with the ID group. This approach allowed us to compare ID + Flare vs. NO ID patients. While ID and Flare represent opposite clinical states, we adopted the classification used in the original study by Spreafico et al. [11], where no significant differences were observed between ID and Flare patients, while both groups showed a significantly different DNAm and transcriptomic profile compared to the intermediate NO ID category.

Linear regression analyses confirmed findings of the main analysis, revealing a significant association between EML and clinical activity, with higher EML values observed in NO ID compared to ID patients at both T_0_ (β = 0.52; 95% CI [0.07–0.97]; p = 0.028) and T_end_ (β = 0.78; 95% CI [0.43–1.13]; p < 0.001). In particular, the estimated effect sizes from the sensitivity analysis were consistent with those of the main analysis, as their confidence intervals substantially overlapped and mutually included the point estimates of the other, supporting the robustness of the observed associations.

Finally, we conducted a differential expression analysis on candidate genes and compared the resulting log_2_FoldChange values with those from our signature. The Spearman correlation observed across the 104 common genes was remarkably strong (ρ = 0.98, p = 1.6 × 10⁻⁶⁹) (**Figure S8**), further validating the consistency of our findings.

**References**

1. Higgins-Chen AT, Thrush KL, Wang Y, Minteer CJ, Kuo P-L, Wang M, et al. A computational solution for bolstering reliability of epigenetic clocks: implications for clinical trials and longitudinal tracking. Nat Aging. 2022;2:644–61.

2. Hillary RF, Marioni RE. MethylDetectR: a software for methylation-based health profiling. Wellcome Open Res. 2020;5:283.

3. Ying K, Liu H, Tarkhov AE, Sadler MC, Lu AT, Moqri M, et al. Causality-enriched epigenetic age uncouples damage and adaptation. Nat Aging. 2024;4:231–46.

4. Jokai M, Torma F, McGreevy KM, Koltai E, Bori Z, Babszki G, et al. DNA methylation clock DNAmFitAge shows regular exercise is associated with slower aging and systemic adaptation. GeroScience. 2023;45:2805–17.

5. Teschendorff AE. A comparison of epigenetic mitotic-like clocks for cancer risk prediction. Genome Med. 2020;12:56.

6. Pelegí-Sisó D, De Prado P, Ronkainen J, Bustamante M, González JR. *methylclock* : a Bioconductor package to estimate DNA methylation age. Robinson P, editor. Bioinformatics. 2021;37:1759–60.

7. Lee S, Emond MJ, Bamshad MJ, Barnes KC, Rieder MJ, Nickerson DA, et al. Optimal Unified Approach for Rare-Variant Association Testing with Application to Small-Sample Case-Control Whole-Exome Sequencing Studies. The American Journal of Human Genetics. 2012;91:224–37.

8. Pedregosa F, Varoquaux G, Gramfort A, Michel V, Thirion B, Grisel O, et al. Scikit-learn: Machine Learning in Python [Internet]. arXiv; 2018 [cited 2024 Sep 25]. Available from: http://arxiv.org/abs/1201.0490

9. Chen T, Guestrin C. XGBoost: A Scalable Tree Boosting System. Proceedings of the 22nd ACM SIGKDD International Conference on Knowledge Discovery and Data Mining [Internet]. San Francisco California USA: ACM; 2016 [cited 2024 Sep 20]. p. 785–94. Available from: https://dl.acm.org/doi/10.1145/2939672.2939785

10. Defazio A, Bach F, Lacoste-Julien S. SAGA: A Fast Incremental Gradient Method With Support for Non-Strongly Convex Composite Objectives [Internet]. arXiv; 2014 [cited 2024 Sep 25]. Available from: http://arxiv.org/abs/1407.0202

11. Spreafico R, Rossetti M, Whitaker JW, Wang W, Lovell DJ, Albani S. Epipolymorphisms associated with the clinical outcome of autoimmune arthritis affect CD4 ^+^ T cell activation pathways. Proc Natl Acad Sci USA. 2016;113:13845–50.
